# Initial Metastatic Site as a Prognostic Factor in Patients With Stage IV Pancreatic Ductal Adenocarcinoma

**DOI:** 10.1097/MD.0000000000001012

**Published:** 2015-06-26

**Authors:** Hyoung Woo Kim, Jong-chan Lee, Kyu-hyun Paik, Yoon Suk Lee, Jin-Hyeok Hwang, Jaihwan Kim

**Affiliations:** From Department of Internal Medicine, Seoul National University College of Medicine, Seoul National University Bundang Hospital, Seongnam-si, Republic of Korea (HWK, J-CL, K-HP, J-HH, JK); and Department of Internal Medicine, Keimyung University School of Medicine, Daegu, Republic of Korea (YSL).

## Abstract

Few studies have evaluated the presence of hepatic or peritoneal metastasis as a prognostic factor in patients with metastatic pancreatic ductal adenocarcinoma (PDAC). This study aimed to elucidate the prognostic value of the initial metastatic, extrahepatic, or hepatic site in patients with metastatic PDAC.

Between January 2007 and December 2013, the medical records of 343 patients with metastatic PDAC treated at Seoul National University Bundang Hospital were retrospectively reviewed. Patients were classified as having extrahepatic metastasis alone (EH), hepatic metastasis alone (LV), and both hepatic and extrahepatic metastasis (BOTH).

The median age was 67 years; 207 patients were men. Patients were classified as having EH (111 patients), LV (106), and BOTH (126). Totally, 212 patients underwent chemotherapy with a FOLFIRINOX (23 patients) or gemcitabine-based regimen (189). On multivariate analysis, an ECOG score ≥2 (hazard ratio [HR]: 3.2, 95% confidence interval [CI]: 2.2–4.5), albumin < 35 g/L (HR: 1.6, 95% CI: 1.1–2.3), C-reactive protein > 10 mg/L (HR: 2.3, 95% CI: 1.6–3.2), neutrophil-lymphocyte ratio > 5 (HR: 1.4, 95% CI: 1.0–2.0), no chemotherapy (HR: 2.0, 95% CI: 1.0–4.1), and metastatic site (LV, HR: 2.1, 95% CI: 1.4–3.1; BOTH, HR: 2.2, 95% CI: 1.6–3.2) were significantly associated with shorter overall survival (OS). Considering the initial metastatic site, the median OS of patients with EH, LV, and BOTH were 7.5 (95% CI: 6.3–8.8), 4.8 (95% CI: 4.1–5.5), and 2.4 (95% CI: 1.9–2.9) months, respectively.

The initial metastatic site is significantly and independently associated with OS in patients with metastatic PDAC, serving as an effective prognostic factor.

## INTRODUCTION

Pancreatic ductal adenocarcinoma (PDAC) is one of the most lethal solid tumors.^1^ At the time of diagnosis, because of local invasion or distant metastasis, 80–90% of patients with PDAC are not candidates for curative surgical resection.^1–3^ Although newer combination chemotherapies such as fluorouracil, leucovorin, irinotecan, and oxaliplatin (FOLFIRINOX) or nab-paclitaxel with gemcitabine were developed as a first-line palliative chemotherapies,^4,5^ the median overall survival (OS) of patients with unresectable PDAC is <1 year.^4–6^ Among patients with unresectable PDAC, there is a significant difference in OS between those with locally advanced or metastatic PDAC. Therefore, the American Joint Committee on Cancer (AJCC) staging system discriminates locally advanced PDAC from metastatic PDAC.^7^

However, there was no further stratification of patients with metastatic PDAC, despite differential prognoses being commonly observed between those with multiple metastases and single metastasis at the time of diagnosis. Recently, several studies suggested that the presence of hepatic or peritoneal metastasis is an independent adverse prognostic factor for OS in these patients.^8–10^ However, it was not evident that there was any difference in prognosis according to the initial metastatic site or tumor burden.^11,12^

In this study, we aimed to determine whether the initial metastatic site could influence the survival and act as a prognostic factor in patients with metastatic PDAC.

## MATERIALS AND METHODS

### Patients

Between January 2007 and December 2013, 441 patients with metastatic PDAC were identified in Seoul National University Bundang Hospital. Among these, 98 patients were excluded for the following reasons: follow-up loss, lack of initial radiologic examination, lack of prior chemotherapy information, no histological confirmation, previous palliative surgery, or multiple cancers. Eventually, 343 patients were enrolled and their medical records were retrospectively reviewed. The study protocol was reviewed and approved by the Institutional Review Board of Seoul National University Bundang Hospital (IRB No.: B-1501-282-108). Informed consent was exempted by the board.

### Definitions

Based on abdominal computed tomography (CT) with or without magnetic resonance imaging (MRI) or positron emission tomography (PET)/CT at initial diagnosis, patients were classified according to the metastatic site as follows: hepatic metastasis alone (LV), extrahepatic metastasis alone (EH), and both hepatic and extrahepatic metastasis (BOTH). Measurement of baseline serum albumin, C-reactive protein (CRP), neutrophil-lymphocyte ratio (NLR), and tumor marker carbohydrate antigen 19-9 (CA19-9) levels were performed during the initial evaluation. Low serum albumin and high CRP was defined as <35 g/L and >10 mg/L, respectively.^4,13–15^ A high NLR was defined as >5.^13,16,17^ A high CA19-9 level was defined as >1000 U/mL.^18,19^ A large primary tumor was defined as more than the median values. OS was defined as the time interval from diagnosis to death, whereas progression-free survival (PFS), in patients who were treated with chemotherapy, was defined as the time interval from diagnosis to disease progression or death, if occurring before documented radiological progression.

### Statistical Analysis

Kaplan–Meier analysis was used to generate survival curves and calculate the median survival times that were compared using the log-rank test. The analysis of the risk factors for death or disease progression was performed using univariate and multivariate Cox proportional hazard regression models. Risk factors were expressed as the hazard ratio (HR). Among the clinical variables included in univariate analysis, those with a two-sided *P*-value of <0.05 were chosen for multivariate analysis with stepwise selection. The distribution of continuous variables is reported as the median and interquartile range (25th–75th percentiles). Comparisons between the subgroups were conducted using one-way ANOVA for continuous variables, whereas a linear by linear association was used to compare noncontinuous variables. A two-sided *P*-value of <0.05 was considered statistically significant. For correlations between variables, the Spearman's correlation coefficient with two-sided significance was used. All statistical analyses were performed using SPSS statistics 21.0 software for Windows (IBM Corporation, Armonk, NY).

## RESULTS

### Patient Characteristics

A total of 343 consecutive patients with stage IV PDAC were enrolled in this study. Baseline patient characteristics are shown in Table 1. According to the initial metastatic site at the time of diagnosis, 111 (32.4%) patients were included in the EH group, 106 (30.9%) in the LV group, and 126 (36.7%) in the BOTH group. Among 111 patients with EH group, 82 (73.9%) patients had peritoneal metastasis. The median patient age at diagnosis was 67 years, and 207 (60.3%) patients were men.

**TABLE 1:**
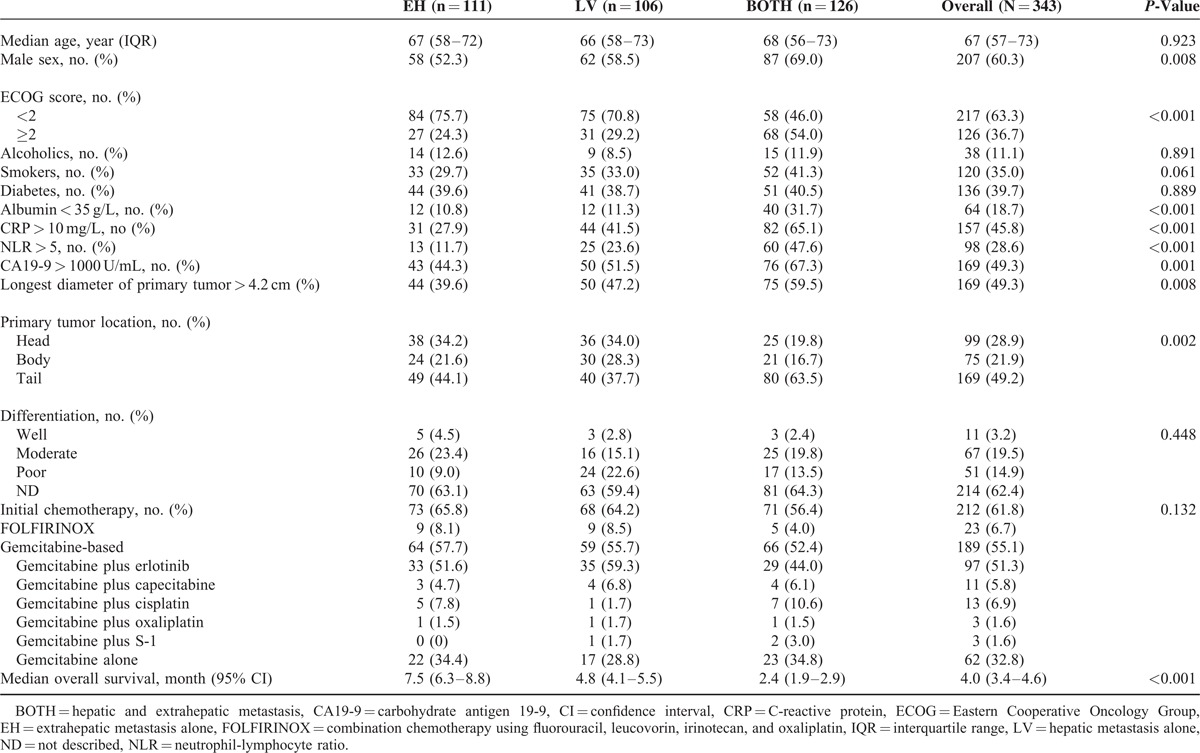
Patient Characteristics

The proportions of male patients was significantly higher in the BOTH group compared with the other groups. Compared with the other 2 groups, patients in the BOTH group had significantly higher Eastern Cooperative Oncology Group (ECOG) scores (≥2), lower albumin, higher CRP, higher NLR, higher CA19-9, larger primary tumors, and more primary tumors located at the pancreatic tail.

A total of 212 (61.8%) patients received initial palliative gemcitabine, gemcitabine-based combination chemotherapy, or FOLFIRINOX chemotherapy. There was no statistically significant difference in the proportion of patients who underwent chemotherapy between the groups (*P* = 0.132), although the majority were administered gemcitabine or gemcitabine-based combination chemotherapy (Table 1).

### Survival Analysis in All Patients

**FIGURE 1:**
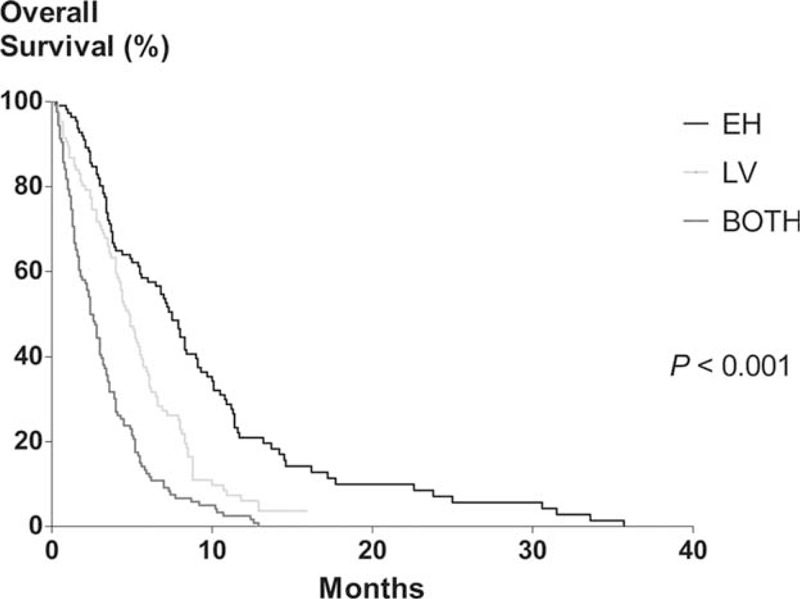
Kaplan–Meier analysis of overall survival in all patients. The median overall survival of EH, LV, and BOTH groups were 7.5 (95% confidence interval [CI], 6.3–8.8), 4.8 (95% CI, 4.1–5.5), and 2.4 (95% CI, 1.9–2.9) months, respectively (*P* < 0.001). EH, extrahepatic metastasis alone; LV, hepatic metastasis alone; BOTH, both hepatic and extrahepatic metastasis.

The median OS for this study population was 4.0 months (95% confidence interval [CI], 3.4–4.6). When survival according to the initial metastatic site was assessed, the median OS of EH, LV, and BOTH groups were 7.5 (95% CI, 6.3–8.8), 4.8 (95% CI, 4.1–5.5), and 2.4 (95% CI, 1.9–2.9) months, respectively (*P* < 0.001; Table 1 and Figure 1). According to univariate analysis, the age, ECOG score, albumin, CRP, NLR, CA19-9, the initial metastatic site, and the initial chemotherapy regimen were all significantly associated with OS. Multivariate analysis revealed that the ECOG score ≥2, low albumin, high CRP, high NLR, initial BOTH or LV metastasis, and no prior chemotherapy were significantly and independently associated with shorter OS (Table 2).

**TABLE 2:**
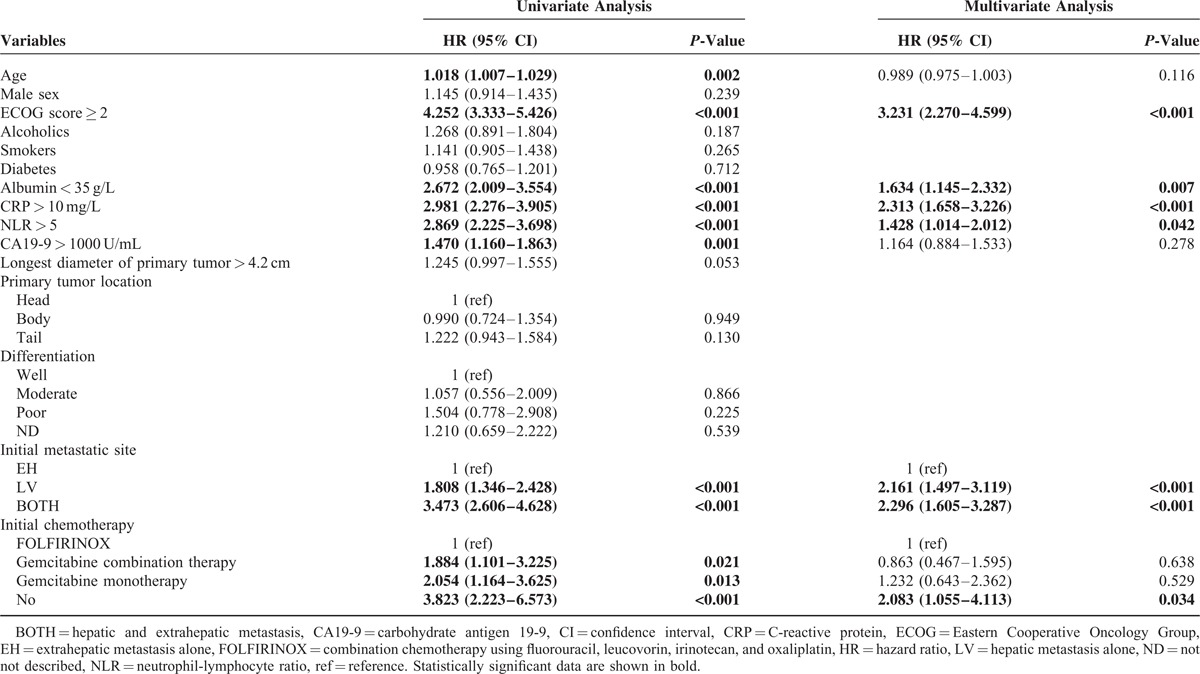
Survival Analysis of All Patients

### Overall and Progression-Free Survival Analysis in Patients Who Underwent Palliative Chemotherapy

**FIGURE 2:**
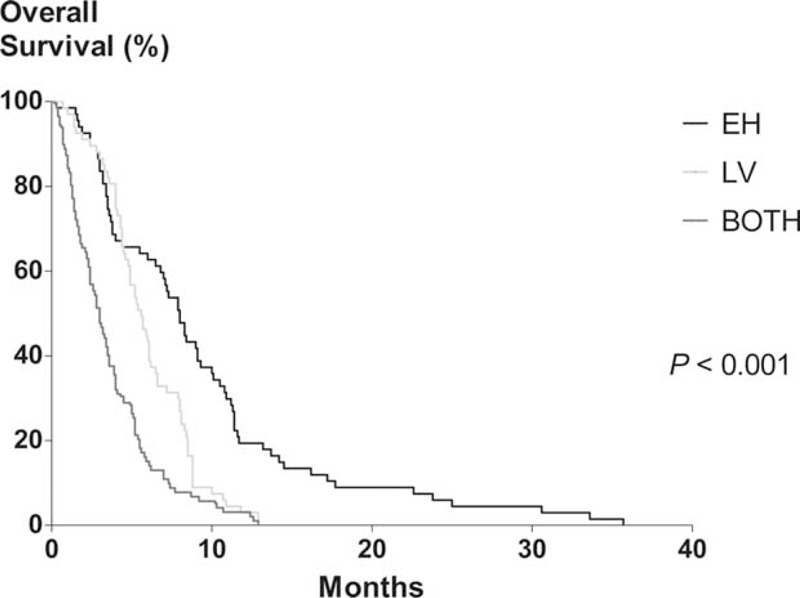
Kaplan–Meier analysis of overall survival in patients who underwent palliative chemotherapy. The median overall survival of EH, LV, and BOTH groups were 8.3 (95% confidence interval [CI], 7.2–9.3), 5.9 (95% CI, 5.2–6.6), and 3.6 (95% CI, 3.0–4.2) months, respectively (*P* < 0.001). EH, extrahepatic metastasis alone; LV, hepatic metastasis alone; BOTH, both hepatic and extrahepatic metastasis.

Among the patients who received initial chemotherapy, the median OS was 5.5 months (95% CI, 4.9–6.1 months). The median OS rates of patients who were administered FOLFIRINOX, gemcitabine-based, or no chemotherapy were 8.8 (95% CI, 3.8–13.8), 5.2 (95% CI, 4.4–6.1), and 2.4 (95% CI, 1.8–2.9) months, respectively. Univariate analysis revealed that the age, ECOG score, albumin, CRP, NLR, CA19-9, the longest diameter of the primary tumor, the initial metastatic site, and the chemotherapy regimen were significantly associated with OS in patients who underwent chemotherapy. Multivariate analysis revealed that an ECOG score ≥2, high CRP, and initial BOTH or LV metastasis were significantly and independently associated with shorter OS in these patients (Table 3). According to the initial metastatic site, the median OS rates of patients who received chemotherapy were 8.3 (95% CI, 7.2–9.3), 5.9 (95% CI, 5.2–6.6), and 3.6 (95% CI, 3.0–4.2) months for the EH, LV, and BOTH groups, respectively (*P* < 0.001; Figure 2).

**TABLE 3:**
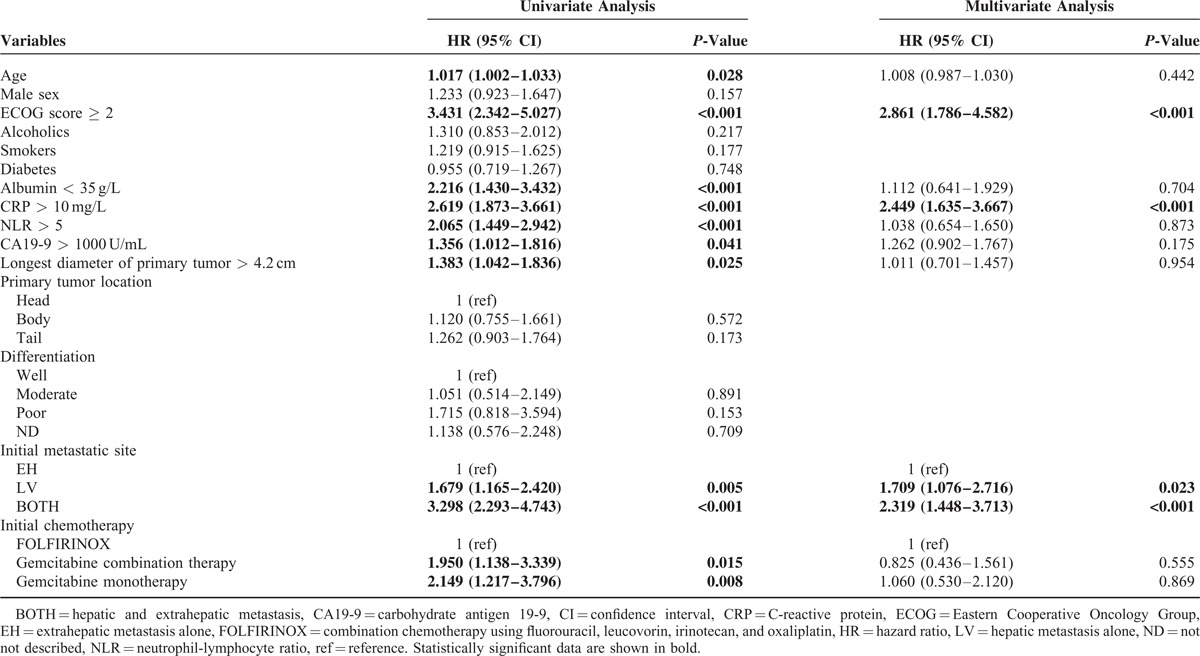
Overall Survival of Patients Treated With Palliative Chemotherapy

**FIGURE 3:**
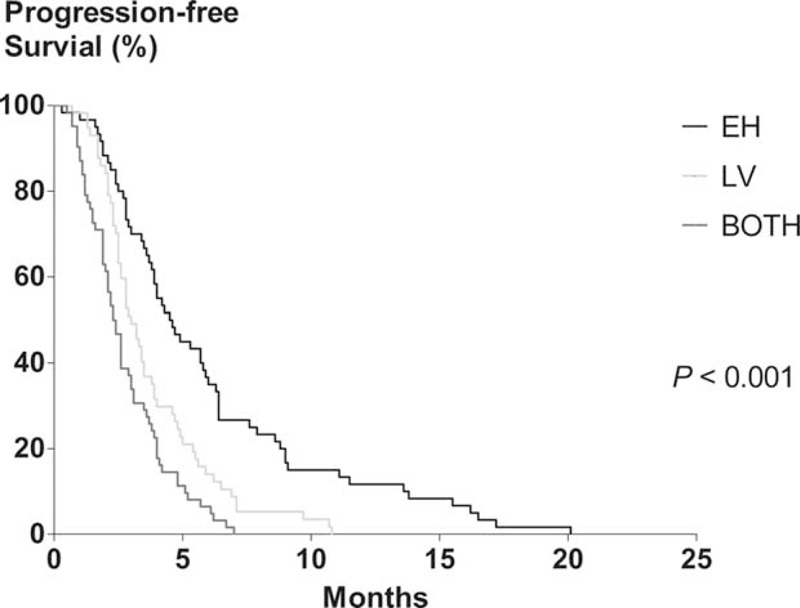
Kaplan–Meier analysis of progression-free survival in patients who underwent palliative chemotherapy. The median progression-free survival of EH, LV, and BOTH groups were 4.9 (95% confidence interval [CI], 3.5–6.3), 3.2 (95% CI, 2.6–3.8), and 2.4 (95% CI, 1.9–2.9) months, respectively (*P* < 0.001). EH, extrahepatic metastasis alone; LV, hepatic metastasis alone; BOTH, both hepatic and extrahepatic metastasis.

In patients who received initial chemotherapy, the median PFS was 3.4 months (95% CI, 2.9–3.9 months). Univariate analysis revealed that the age, ECOG score, albumin, CRP, NLR, the longest diameter of the primary tumor, initial BOTH or LV metastasis, and the chemotherapy regimen were all significantly associated with PFS. Multivariate analysis revealed that an ECOG score ≥2, high CRP, and initial BOTH or LV metastasis were significantly and independently associated with shorter PFS (Table 4). When PFS according to the initial metastatic site was analyzed, the median PFS of EH, LV, and BOTH groups were 4.9 (95% CI, 3.5–6.3), 3.2 (95% CI, 2.6–3.8), and 2.4 (95% CI, 1.9–2.9) months, respectively (*P* < 0.001; Figure 3).

**TABLE 4:**
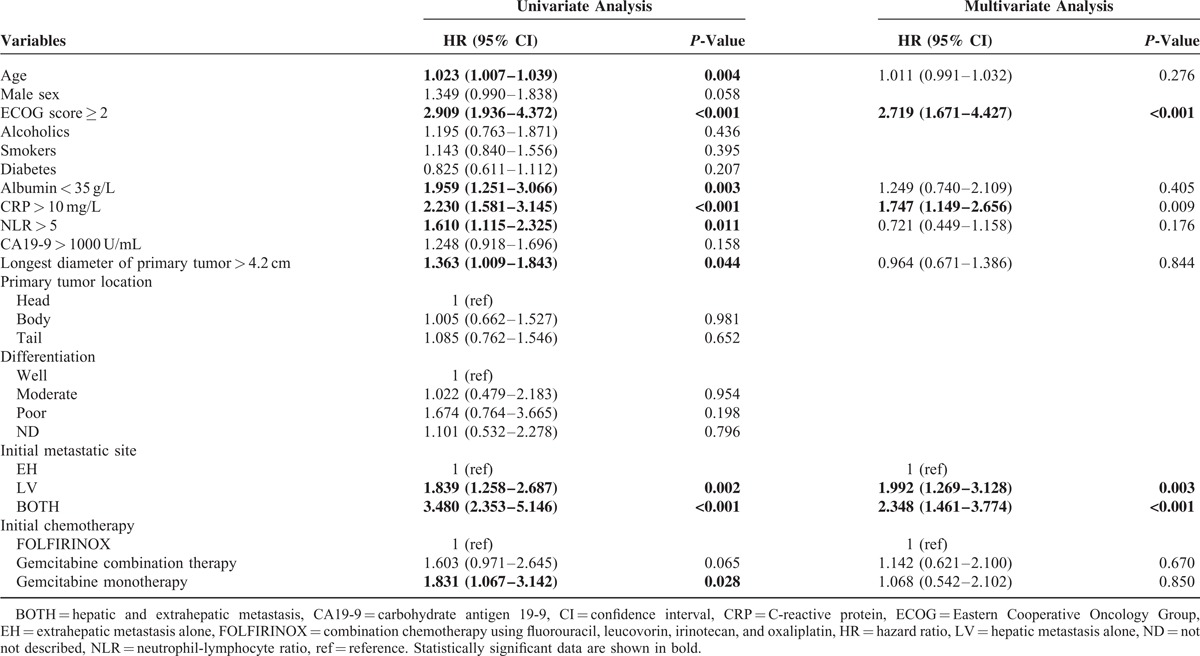
Progression-Free Survival of Patients Treated With Palliative Chemotherapy

## DISCUSSION

Among patients with unresectable PDAC, the prognosis is different between locally advanced and metastatic cancer. However, there was no further stratification in patients with metastatic PDAC which is the most common stage at the time of diagnosis. In such background, we attempted to investigate survival according to the initial metastatic site and determine the prognostic role of the metastatic site in patients with metastatic PDAC.

In the present study, patients with concomitant extrahepatic and liver metastasis showed the worst prognosis compared to those with a single metastatic site. The patients in the BOTH group had generally less desirable clinical characteristics than those in the other 2 groups, including higher ECOG performance scores, poorer nutritional status (albumin level), more severe inflammation (CRP and NLR), higher CA19-9 levels, and larger primary tumor sizes. As a result, the OS of those in the BOTH group was significantly shorter compared with the other 2 groups. Similarly, the above-mentioned poor prognostic factors were more apparent in the LV group compared with the EH group, which was also reflected in the significantly shorter OS of the LV group compared with the EH group. In summary, patients with initial EH metastasis had significantly better OS compared with those in the other 2 groups. Multivariate analysis confirmed that the initial metastatic site was significantly and independently associated with the survival outcome.

PDAC spreads widely to the various organs and tissues, especially the liver and peritoneum.^20^ Recent studies have suggested a prognostic index or model for OS based on hepatic or peritoneal metastasis, serum CRP, serum albumin, abdominal and/or back pain, or the performance status.^8–10^ Although these models could predict prognosis in patients with PDAC, all had potential limitations because of the complexity and subjectivity of some of the variables such as abdominal pain or performance status. In contrast, determining the initial metastatic site as extrahepatic, hepatic, or both, as seen here, was relatively simple and would be easy to apply as a prognostic factor in patients with metastatic PDAC. However, there was no further difference of OS between patients with and without peritoneal metastasis in this study.

Recently, FOLFIRINOX or a combination of gemcitabine and nab-paclitaxel has been used as standard regimens in patients with metastatic PDAC who have a good performance status.^4,5^ Prior to that, single gemcitabine, gemcitabine plus erlotinib, or gemcitabine plus capecitabine were commonly administered for palliative chemotherapy in patients with metastatic PDAC.^21–23^ The present study demonstrated that a lack of palliative chemotherapy was a significant and independent risk factor for poor OS compared with patients who received FOLFIRINOX therapy. However, gemcitabine-based therapy did not have any beneficial effect on the OS of these patients. This is possibly because, even as a first-line treatment, gemcitabine-based chemotherapy has marginal benefits compared to best-supportive care,^21^ and therefore, in comparison to FOLFIRINOX, a significant lack of effect of gemcitabine-based treatment on OS is hardly surprising.

The present study has limitations. First, because of its retrospective design, the initial metastatic site may be underestimated because liver MRI (53 patients, 15.5%), PET/CT (120 patients, 35.0%), chest CT (89 patients, 25.9%), or bone MRI (31 patients, 9.0%) were only performed in 188 patients whose abdominal CT results were not satisfactory. However, it should be considered that such imaging studies are not routinely recommended now. Second, the system we applied for metastasis classification was not proportional to the actual tumor burden, but the classifications were objective and easy to apply, and there was an almost equal distribution of patient number among the groups in the study. Finally, there were more than 60% of cases of which pathologic differentiation was not described because of small biopsy tissue sample.

In conclusion, patients with BOTH had the worst prognosis. Patients with EH had better prognosis than those with hepatic metastasis. Therefore, the initial metastatic site is an effective and easily applicable prognostic factor for patients with metastatic PDAC.
